# Case Report: Transcatheter valve implantation in a 13-year-old teenager with critical aortic stenosis and Singleton–Merten syndrome

**DOI:** 10.3389/fcvm.2025.1506887

**Published:** 2025-05-08

**Authors:** T. M. Pervunina, O. B. Irtyuga, T. L. Vershinina, M. V. Babakekhyan, A. A. Lyapunova, A. A. Kostareva, D. D. Zubarev, A. V. Gorbatykh, A. A. Prokhorikhin, E. V. Grekhov, E. S. Vasichkina, E. V. Shlyakhto

**Affiliations:** V.A. Almazov National Medical Research Centre, Saint Petersburg, Russia

**Keywords:** TAVI, Singleton–Merten syndrome, teenager, clinical case, aortic stenosis

## Abstract

This article presents a rare case of transcatheter aortic valve implantation (TAVI) in a critically ill teenager with Singleton–Merten syndrome. The procedure was successful, and the patient was discharged home without complication. To the best of our knowledge, this is the first report combining disease, age, and TAVI.

## Introduction

Singleton–Merten syndrome is a rare autosomal dominant interferonopathy caused by a mutation in the DDX58 gene and characterized by dental dysplasia, aortic calcification, skeletal anomalies, glaucoma, psoriasis, osteoporosis, and other manifestations (1–4). Moreover, a group of researchers (Parra-Izquierdo et al.) demonstrated a correlation between male sex and the frequency of increased activity of IFN-α-cytokine, characterized by pro-inflammatory and pro-osteogenic effects (5). The syndrome was described for the first time in 1993 by Singleton and Merten in two patients with dental anomalies, osteoporosis of the distal extremities, bilateral glaucoma, and severe calcification of the aortic valve (AV) and aortic arch, which led to death.

Based on the type of genetic rearrangement, there are two known main types of this disease. Singleton–Merten syndrome type 1 is associated with a mutation in the IFIH1 protein gene (5–11).

Singleton–Merten syndrome type 2 is caused by a mutation in the DDX58 gene. According to global medical literature, this genetic variant of the disease is characterized by ophthalmological (glaucoma) and skin manifestations (psoriasis), and has a more favorable prognosis compared to type 1 Singleton–Merten syndrome (12, 13). In addition, it was established that increased expression of the DDX58 gene leads to an increase in the production of pro-inflammatory cytokines, which contributes to increased calcification of the aorta and AV, and, consequently, to aortic stenosis development (14, 15).

At present, methods to increase gene expression are being actively studied (16, 17), however, surgical intervention remains the main method to treat severe symptomatic valvular heart disease. This is normally done through open cardiac surgery with AV replacement or minimally invasive surgery—transcatheter aortic valve implantation (TAVI) (18).

The literature describes very few cases of TAVI being performed on young patients with congenital heart defects, the development of AV insufficiency after a previous Ross operation, or on children with severe AV pathology and terminal heart failure (HF) (19–22). In August 2022, a 10-year retrospective study (2010–2020) was published in the American Journal of Cardiology comparing the outcomes and prognosis of patients undergoing open surgical intervention for AV insufficiency (*n* = 60) and patients after TAVI (*n* = 17). It is noteworthy that the patients' ages varied from 10 to 21 years. The incidence of stroke within 6 months after surgery, repeat hospitalization within 30 days, and all-cause death did not differ between groups, while the time spent in the intensive care unit and whole hospital stay were higher in the patients who underwent open surgery (23).

As for Singleton–Merten syndrome, there is only one published case of a 21-year-old woman who underwent TAVI due to severe aortic regurgitation (24). The postoperative period was uneventful and the patient was discharged 4 days after the procedure.

Therefore, considering the use of TAVI in pediatric and young patients with severe AV pathology, including that caused by hereditary interferonopathy, is currently extremely relevant.

## Featured case

In November 2022, a 13-year-old boy was admitted to the Pediatric Cardiology Department of the V.A. Almazov National Medical Research Center with complaints of increased fatigue, shortness of breath during minimal physical exertion (PE), chest pain unrelated to physical activity, and ankle swelling that had persisted since June 2022. In addition, the patient noted the appearance of a cough in the prone position and had slept with a raised headboard. There was a single episode of syncope in 2020 without an obvious trigger.

Singleton–Merten syndrome type 2 (mutation in the DDX58 gene) was genetically confirmed in 2021 and manifested with an incomplete clinical picture: cardiovascular diseases with calcification of the aorta and aortic and mitral valves (MV), osteoporotic manifestations, dental and skeletal anomalies, psoriatic skin lesions, and bilateral glaucoma. Neither parent carried this mutation according to genetic tests. At the age of 2 years, bilateral glaucoma was diagnosed and surgical treatment was performed. At the age of 9, psoriasis was diagnosed; at the age of 10, the patient complained of stiffness in the ankle and interphalangeal joints of the upper and lower extremities, periodic swelling and hyperemia of the interphalangeal joints, and pain in the heel bones when walking. According to x-ray scans of his hands, there were signs of metacarpophalangeal joints arthritis in both hands, osteoporosis of the epiphyses of the ulnar, radial, and carpal bones and phalanges and metacarpals, followed by the formation of multiple flexion contractures of the elbow, radiocarpal, ankle, and interphalangeal joints. At the age of 8, a systolic murmur was detected in the left second intercostal space, and the subsequent examination confirmed the presence of a congenital bicuspid aortic valve with moderate stenosis. At that time, the patient received no therapy. During the following years, the patient’s condition progressively worsened due to calcification of the aorta and aortic valve, an increase in the AV pressure gradient, and the development of heart failure. Despite the clinical indications, the parents consistently refused the offered surgical intervention. The patient was discharged on medical therapy.

**Figure 1:**
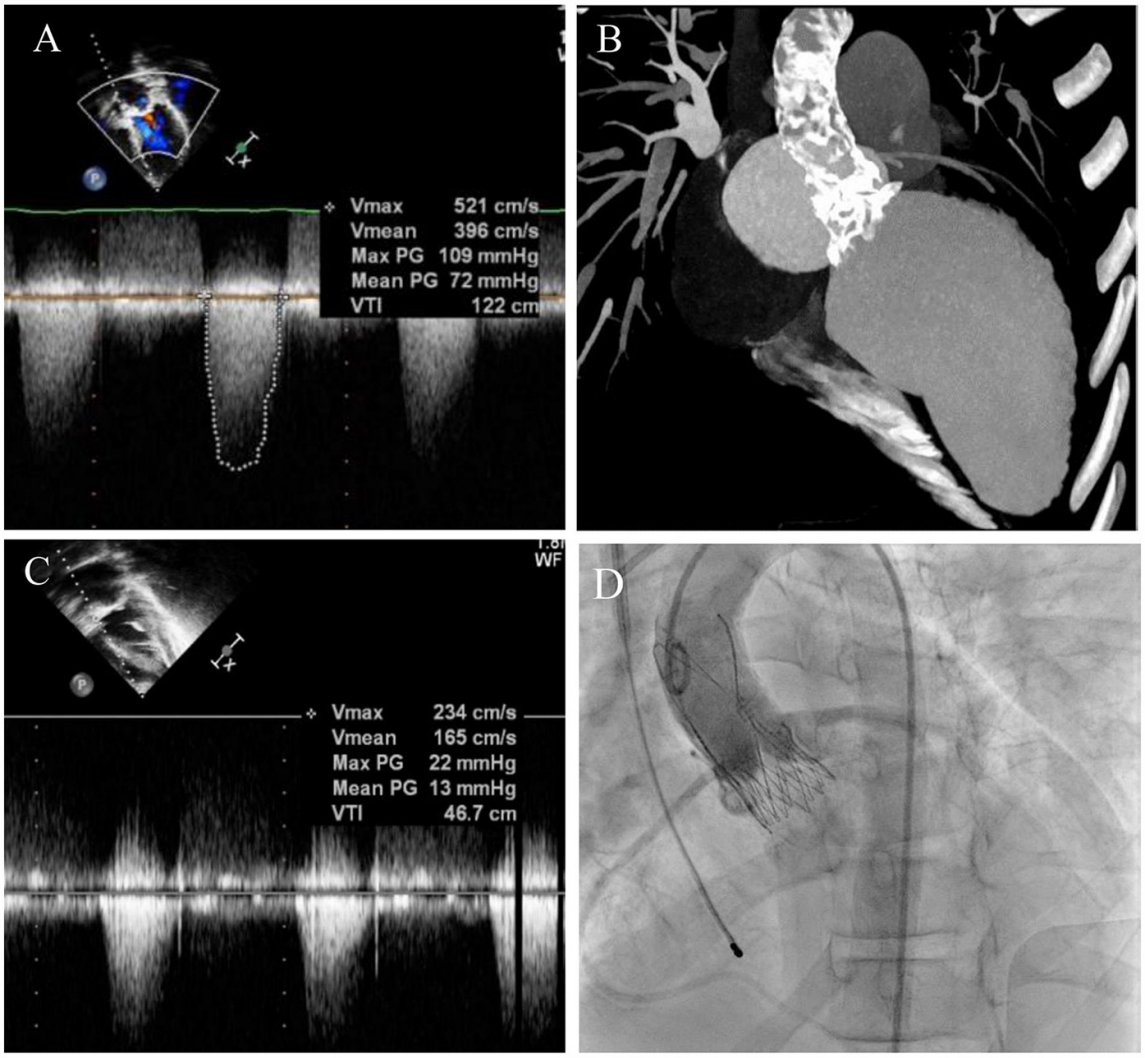
Pre-TAVI assessment and postprocedural result. **(A)** Preoperative echocardiography showing V_max_ of 5.2 m/s and mean aortic pressure gradient of 72 mmHg. **(B)** CT-angiography demonstrating severe calcification of the aortic root and ascending aorta. **(C)** Postoperative transthoracic echocardiography confirmed a decrease in V_max_ and mean AV gradient. **(D)** Postimplantation aortogram.

The patient was then admitted with New York Heart Association (NYHA) Class IV heart failure. A cardiac echocardiogram revealed enlargement of the left heart chambers [left ventricular (LV) end-diastolic dimension (EDD) 53 mm, z-score of 1.92; LV end-diastolic volume (EDV) of 116.6 ml/m^2^], decreased myocardial contractility of the left [left ventricular ejection fraction (LVEF) of 20%–22% using Simpson’s method] and right ventricles [tricuspid annular plane systolic excursion (TAPSE) of 9 mm], critical mixed aortic valve disease [aortic valve area (AVA) of 0.8 cm^2^, peak AV gradient of 109 mmHg, mean AV gradient of 72 mmHg, maximum velocity (V_max_) of 5.2 m/s, aortic regurgitation of 2°–3°, and vena contracta of 5–8 mm], moderate mixed mitral valve disease (V_max_ of 1.64 m/s, mean MV gradient of 10 mmHg, and moderate mitral regurgitation), and pericardial effusion of 13–14 mm with no evidence of collapse (Figure 1A). In addition, left ventricular hypertrophy with posterior wall (13 mm, z-score of 4.98) and septum (19 mm, z-score of 5.51) thickening was diagnosed.

Based on the assessment results and the ineffectiveness of conservative treatment, a surgical intervention with the Konno–Rastan procedure and MV replacement was considered as the first option. However, due to severe calcification of the left ventricular outflow tract (LVOT), aortic root, and ascending aorta revealed on multidetector computed tomography (MDCT) (Figure 1B); decreased severe biventricular heart failure; and the high risk of open surgery, transcatheter aortic valve replacement was considered the only treatment option. After collecting signed informed consent from the parents, the TAVI procedure was conducted.

The intervention was performed with no complications. An ACURATE neo S 23-mm transcatheter bioprosthesis (Boston Scientific, USA) was implanted in an aortic position through a 14 Fr iSleeve transfemoral sheath (Boston Scientific, USA). Control angiography showed minimal paraprosthetic regurgitation and no signs of coronary ostia obstruction (Figure 1C). The operative access was sutured using a transcutaneous technique with Proglide + Angioseal (Abbott, USA; Terumo, Japan).

The patient was then transferred to the intensive care unit for observation and was discharged to the ward the following day. He remained in sinus rhythm and did not require a pacemaker postoperatively. The patient received standard double antiplatelet therapy (DAPT), however, recurrent nasal bleedings occurred that induced thrombocytopenia, and DAPT was reduced to 50 mg of acetylsalicylic acid and 25 mg of clopidogrel. He also continued receiving heart failure therapy, resulting in the resolution of the edema, pericardial effusion, ascites, and hepatomegaly.

The post-TAVI echocardiography showed a well-functioning aortic valve (mean gradient of 12 mmHg and V_max_ of 2.1 m/s) with trivial paravalvular leakage and no pericardial effusion. The parameters of both ventricles demonstrated substantial improvement with LVEF increased to 30% and TAPSE to 21 mm (Figure 1D). The moderate mitral valve disease apparent before the procedure remained unchanged.

After rehabilitation, the patient was discharged home within 22 days. The 4-month follow-up showed improved tolerance to PE and compensation HF symptoms (NYHA II class). A cardiac echocardiogram also demonstrated continuous restoration of ventricular function (LVEF of 50% and TAPSE of 25 mm) and a preserved aortic valve (mean gradient of 4 mmHg and V_max_ of 1.47 m/s).

## Case discussion

Our case presents a young patient with a rare genetic disease in whom the benefits of a transcatheter strategy allowed for myocardial recovery under critical circumstances. Despite all the concerns regarding the performance of TAVI in children with Singleton–Merten syndrome and critical heart failure, the provided procedure significantly improved the boy's condition.

There are limited reports describing surgical strategies in children with childhood-onset disorders featuring severe aortic stenosis with a porcelain aorta. We found no patients with Singleton–Merten syndrome and only a few with Gaucher disease, an autosomal recessive lysosomal storage disorder, who successfully underwent aortic replacement (25). However, the clinical condition of the teenager and the severity of the calcification made the surgical approach unacceptably risky.

The postoperative evaluation revealed rapid restoration of ventricular performance, demonstrating the tremendous contractility reserve in young patients. Future concerns regarding long-term prognosis depend on the effectiveness of Janus kinase inhibitor therapy and the durability of the TAVI prosthesis. In our opinion, the expected progression of aortic calcification leaves only the TAVI-in-TAVI option for future intervention. We will continue to monitor the teenage patient.

## Conclusion

To our knowledge, this is the first case of TAVI in an adolescent affected by Singleton–Merten syndrome. We consider this strategy to be a life-saving option for a critically ill patient and a potential bridge to definitive treatment when the patient reaches adulthood. Due to its rarity, we hope this case will help in the understanding and management of this rare and complex disease.

## Data Availability

The original contributions presented in the study are included in the article/Supplementary Material, further inquiries can be directed to the corresponding author.
